# The Current Evidence for Vegetarian and Vegan Diets in the Management of Type 2 Diabetes

**DOI:** 10.1016/j.advnut.2024.100296

**Published:** 2024-09-30

**Authors:** Sabrina Schlesinger, Lukas Schwingshackl

**Affiliations:** 1 Institute for Biometrics and Epidemiology, German Diabetes Center, Leibniz Center for Diabetes Research at Heinrich Heine University Düsseldorf, Düsseldorf, Germany; 2 German Center for Diabetes Research (DZD), Partner Düsseldorf, München-Neuherberg, Germany; 3 Institute for Evidence in Medicine, Medical Center – University of Freiburg, Faculty of Medicine, University of Freiburg, Freiburg, Germany

Because of the high prevalence of persons with type 2 diabetes, the management of this disease is of significant interest to researchers, healthcare professionals, and those affected by the condition. As lifestyle factors, including diet, contribute to the development of type 2 diabetes, a substantial body of research also focuses on managing this disease through lifestyle modifications, particularly dietary approaches. In this context, recent evidence has highlighted that different dietary approaches, including plant-based diets, offer potential benefits for managing type 2 diabetes [1].

Building on this body of evidence, Guest et al. [2] conducted a systematic review and mete-analysis on randomized controlled trials (RCTs) investigating the effects of vegetarian and vegan diets on a wide spectrum of outcomes in type 2 diabetes management, commissioned by the Academy of Nutrition and Dietetics, the Academy of Nutrition and Dietetics Foundation, and the Academy of Nutrition and Dietetics Vegetarian Nutrition Dietetic Practice Group. The outcomes ranged from glycemic control, insulin markers, and BMI (kg/m^2^), to blood lipids, reduction of glucose-lowering medications, and health-related quality of life. The findings indicate that vegetarian or vegan diets are likely effective in improving Hemoglobin A1c (HbA1c), reducing BMI, and potentially lowering the dosage of diabetes medications. For outcomes, such as blood lipid concentrations, the effects pointed to similar benefits, although the results were imprecisely estimated, as indicated by wide 95% confidence intervals. For outcomes such as insulin sensitivity or health-related quality of life, the evidence is very uncertain, because findings are based on only few or single RCTs.

The findings of this methodologically sound evidence synthesis support the initial steps of the current dietary guidelines taken by leading diabetes organizations to include plant-based diets, such as vegetarian diets, in type 2 diabetes management [3,4]. Additionally, the systematic review by Guest et al. (Geust el al.) highlights the need for future research directions in this area. In total, only 7 RCTs were identified, and for some outcomes, only 3–5 RCTs could be included in the meta-analyses. For certain specific outcomes, such as insulin sensitivity or non-HDL cholesterol, only single study findings were available. Despite the small number of RCTs, the number of participants included in the RCTs was also low (in total 770 persons, with a maximum of 383 persons in the meta-analyses). Whether vegetarian and vegan diets differ in their effectiveness compared with each other in managing type 2 diabetes remains uncertain. Additionally, the question whether these dietary approaches are more beneficial compared with other dietary interventions also remains open, because there were differences in the comparison groups across the studies. Some RCTs compared vegetarian diets with usual eucaloric diets, whereas others used, for example, energy restricted diets as the comparator, which introduces differences in energy intake, although subgroup analyses on energy restriction confirmed the results of the main analysis. However, because of the small number of studies and participants, subgroups analyses were only feasible for few outcomes (HbA1c, BMI, and LDL cholesterol) (Geust el al.). Focusing on precision diabetology, the hypothesis that individuals with different diabetes phenotypes may respond differently to dietary interventions, including vegetarian or vegan diets, remains an area of interest.

Beyond that, we learned from observational studies, that plant-based diets are associated with lower risk of type 2 diabetes. The evidence points out that persons adhering to plant-based diets, or follow a vegetarian or vegan diet are less likely to develop type 2 diabetes in the future, even after adjusting for several important confounding factors, such as age, sex, socioeconomic factors, other lifestyle factors, and BMI [5]. Notably, the diet quality of the plant-based regimes is crucial, with evidence indicating a decreased risk for high-quality plant-based diets, and even an increased risk of type 2 diabetes for those following low-quality plant-based diets [6]. However, the evidence on plant-based diets and the progression of type 2 diabetes, including the development of diabetes-related complications, such as cardiovascular diseases, is very limited. Available evidence suggests that foods from plant sources (e.g., whole grains, vegetable, or nuts) are associated with reduced premature death in persons with diabetes [7]. A recent study showed that adherence to a plant-based diet might be associated with lower inflammatory biomarkers in persons with type 2 diabetes, and the risk might differ between diabetes subtypes [8]. However, more research is needed on the impact of vegetarian or vegan diets and clinical important endpoints across diabetes subgroups, considering the quality of the diet and its food components.

Moreover, in addition to the potential health benefits for humans, plant-based diets also contribute to planetary health by reducing greenhouse gas emission, lowering the use of land and water, and preserving biodiversity [9]. However, when adopting more restrictive forms, such as a vegan diet, it is crucial to critically consider nutrients that may be more challenging to obtain solely from plant sources, such as vitamin B12, iron, omega-3 fatty acids, or proteins, to maintain overall health [10].

In conclusion, the current evidence suggests that plant-based diets are a feasible option for managing cardiometabolic health, particularly in those with type 2 diabetes. For individuals who do not wish to completely eliminate meat or animal products, even a reduction in these products with a shift toward healthier plant-based options may already be beneficial for health.

## Author contributions

Both authors read and approved the final manuscript.

## Conflicts of interest

LS is an Associate Editor of Advances in Nutrition. SS reports no conflicts of interest.
